# Dual active layer a-IGZO TFT via homogeneous conductive layer formation by photochemical H-doping

**DOI:** 10.1186/1556-276X-9-619

**Published:** 2014-11-18

**Authors:** Seung-Ki Jeong, Myeong-Ho Kim, Sang-Yeon Lee, Hyungtak Seo, Duck-Kyun Choi

**Affiliations:** 1 Department of Materials Science and Engineering, 222 Wangsimni-Ro, Seongdong-Gu, Hanyang University, Seoul 133-791, Republic of Korea; 2 Department of Materials Science and Engineering and Department of Energy System Research, Ajou University, 206 Worldcup-ro, Yeongtong-Gu, Suwon 443-739, Republic of Korea

**Keywords:** TFT, UV irradiation, Photochemical doping, Hydrogen donor, a-IGZO, Dual active layers

## Abstract

In this study, InGaZnO (IGZO) thin film transistors (TFTs) with a dual active layer (DAL) structure are fabricated by inserting a homogeneous embedded conductive layer (HECL) in an amorphous IGZO (a-IGZO) channel with the aim of enhancing the electrical characteristics of conventional bottom-gate-structure TFTs. A highly conductive HECL (carrier concentration at 1.6 × 10^13^ cm^-2^, resistivity at 4.6 × 10^-3^ Ω∙cm, and Hall mobility at 14.6 cm^2^/Vs at room temperature) is fabricated using photochemical H-doping by irradiating UV light on an a-IGZO film. The electrical properties of the fabricated DAL TFTs are evaluated by varying the HECL length. The results reveal that carrier mobility increased proportionally with the HECL length. Further, a DAL TFT with a 60-μm-long HECL embedded in an 80-μm-long channel exhibits comprehensive and outstanding improvements in its electrical properties: a saturation mobility of 60.2 cm^2^/Vs, threshold voltage of 2.7 V, and subthreshold slope of 0.25 V/decade against the initial values of 19.9 cm^2^/Vs, 4.7 V, and 0.45 V/decade, respectively, for a TFT without HECL. This result confirms that the photochemically H-doped HECL significantly improves the electrical properties of DAL IGZO TFTs.

## Background

Recently, there has been a growing need in the next-generation display industry for the development of new channel thin film transistors (TFTs) in order to implement active-matrix organic light-emitting diode (AMOLED) displays with a large area, rapid information transmission, high resolution, and high frame rates [1]. Among the various candidates for new channel TFTs, oxide TFTs are receiving great attention owing to their several advantages such as high electron mobility, high on/off ratio, low leakage current, and good uniformity even when deposited at low temperatures. In particular, amorphous InGaZnO (a-IGZO) TFT is emerging as the most viable option among oxide TFTs for use in the next-generation display industry, as it provides good uniformity as an amorphous TFT suitable for large displays and has a relatively high mobility of over 10 cm^2^/Vs [2-4].

A-IGZO TFT has higher electron mobility than existing silicon-based TFTs, which are less amorphous. However, for OLED pixels that are utilized in large AMOLED TVs, high current is generally required in order to emit light through electrical current injection. In addition, there is a great demand for TFTs with electron mobility higher than 30 cm^2^/Vs in order to realize the required display resolutions and pixel circuits [5]. To fulfill these requirements, extensive research has been conducted in an attempt to enhance the electron mobility of a-IGZO via different methods, including oxygen vacancy control, [6,7] hydrogen annealing, [8,9] use of a Ca capping layer, [10] sputtering power control, [6] and by combining various oxide components [11,12]. However, oxygen vacancy control - one of the most commonly used methods to enhance the electrical conductivity of oxide semiconductors - may improve their conductivity, but the conductivity range in which this method is applicable is very limited. Hydrogen annealing increases the number of oxygen vacancies by artificially creating a reducing atmosphere and makes it possible to realize high electrical conductivity; however, this method requires a lengthy thermal process carried out above 300°C and is very limited in its applicability as it may damage surrounding devices through rapid hydrogen diffusion. In addition, attempts have been made to enhance the electron mobility of a-IGZO by forming an additional Ca capping layer on top of the active layer to induce the reduction of a-IGZO and increase the number of oxygen vacancies. However, this method encountered problems such as restrictions by the surrounding environment due to vulnerability of Ca to oxygen or moisture in the atmosphere and the complexity of the fabrication process in forming additional layers [10]. Further, sputtering power control and the combination of various oxide materials have reproducibility problems, since target, equipment, and process requirements have not been standardized.

With this background, many researches have been conducted on dual active layer (DAL) TFTs to enhance their mobility by forming additional materials with high electrical conductivity on either the top or bottom of active layers. Kim et al. [13] achieved improvement in mobility by depositing an additional layer of a Mo thin film on the active layer. However, the mobility did not increase noticeably (2.5 to 4.7 cm^2^/Vs), and the transparency decreased with increasing metal length. In another study, a high electrical conductivity oxide such as ITO was embedded in the channel path of the active layer to enhance both transmittance and mobility [14]. However, in this method, an interface is generated between the active layer and oxide material owing to the formation of a heterojunction [15], and this interface formation may degrade the electrical properties of the TFTs.

Hence, clearly distinguished from all the above-discussed previous reports, the present study was focused on high-performance DAL TFTs fabricated using a strikingly convenient optical method called ‘permanent photochemical doping,’ which can uniformly enhance electrical properties by the effective H donor incorporation in the channel, in an attempt to resolve the issues encountered by existing DAL TFTs. Among the available oxide semiconductors, a-IGZO with excellent electrical properties was used for fabricating the TFT active layer, and a homogeneous embedded conductive layer (HECL) obtained through photochemical doping at room temperature was embedded in the channel path to enhance electrical mobility. In addition, the electrical characteristics of the fabricated DAL TFTs were evaluated by varying the HECL length within a certain range to be shorter than the length of the active layer to investigate the effect of HECL on the channel path.

## Methods

Bottom-gate-structure TFTs with an embedded highly conductive a-IGZO path in a-IGZO active layer were fabricated on a glass substrate. A 100-nm-thick Mo gate electrode was deposited using DC sputtering at room temperature and patterned out using wet etching. This was followed by the deposition of a 100-nm-thick SiO_2_ gate insulator at 300°C using plasma-enhanced chemical vapor deposition. For HECL formation, a 20-nm-thick a-IGZO layer was deposited on PR-patterned SiO_2_ substrates with a width of 50 μm and lengths of 20, 40, and 60 μm by RF sputtering at room temperature by using a single oxide target. During a-IGZO deposition, an ambient atmosphere of Ar:O_2_ with a ratio of 90:10 was maintained at 5 mTorr. For HECL formation, after a lift-off process, patterned a-IGZO layers were steadily irradiated by deep-UV light (with main wavelengths of 185 and 254 nm) for 240 min. An additional 20-nm-thick active layer of a-IGZO was then deposited at an RF power of 40 W and room temperature. The 50-μm-wide and 80-μm-long a-IGZO active layer was patterned again using a lift-off process. For fabricating source and drain electrodes, 100-nm-thick Mo layers were deposited using DC sputtering at room temperature.

The electrical properties of the fabricated TFTs were evaluated via current–voltage (*I*-*V*) measurements performed using an Agilent E5270B parameter analyzer (Agilent Technologies, Inc., Santa Clara, CA, USA). The electrical properties of the HECL itself were analyzed using the Van der Pauw method by Hall measurement (Ecopia HMS 3000, Ecopia, Anyang, Korea) at room temperature. The chemical compositions of the oxide thin films were analyzed using X-ray photoelectron spectroscopy (XPS; Theta probe base system, Thermo Fisher Scientific Co., Seoul, South Korea). The energy resolution for each point was 0.05 eV, and the peak energy was self-calibrated to C1s and O1s reference peak states. The optical absorption data were measured by a rotating compensator-enhanced SE device with a spectral resolution of 15 meV and were analyzed using a three-phase optical model.

## Results and discussion

**Figure 1:**
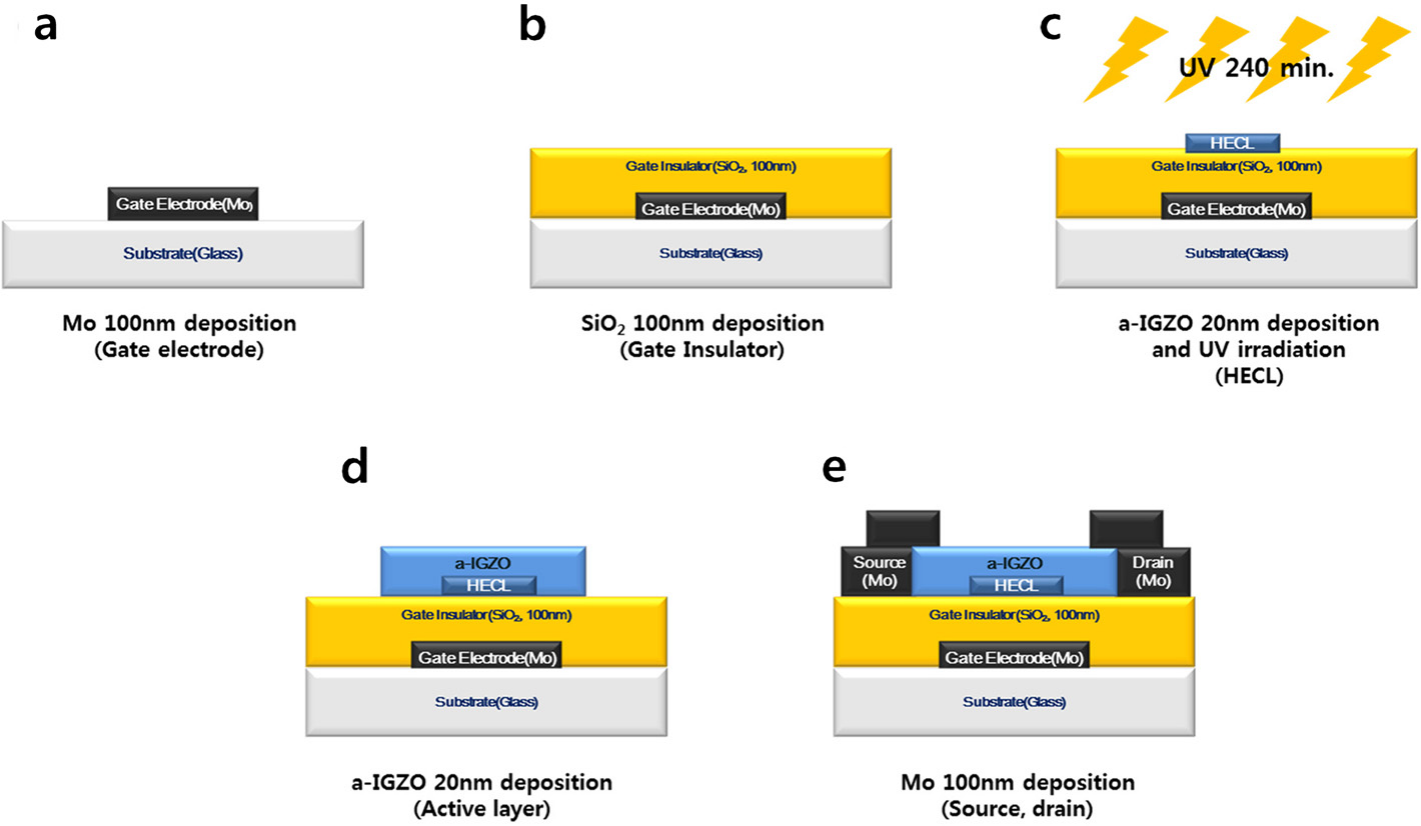
**A schematic representation of the fabrication process for DAL TFTs in the process order from (a-e).** The placement of the 20-nm-thick HECL was selected by considering the channel path formed between the actual source and drain. To confirm the direct influence of HECL, the HECL length was varied within a range to be shorter than the channel path (0, 20, 40, and 60 μm).

We fabricated DAL TFTs by utilizing the bottom-gate-structure TFT that is typically used in displays because of its superior surface properties and also because it does not require a separate shield layer. Figure 1 shows a schematic of the fabrication process for a DAL TFT with a channel width and length of 50 and 80 μm, respectively. The DALs were fabricated by forming an HECL below the active layer; the placement of the HECL was selected by considering the actual channel path formed between the source and drain. When using a DAL structure design in which the DAL is formed by employing other highly conductive materials, the electronic band structure of these materials should be considered. This is because injected carriers from the source electrode can be blocked by the formation of a barrier and their movement toward the drain electrode can be hindered, which will lead to low mobility [14]. However, under such a situation of barrier formation, the electrical properties are expected not to degrade, since the photochemically processed highly conductive HECL and the active layer are formed from the same material, namely, a-IGZO, and thus, they form a pseudo-homogeneous junction.

**Figure 2:**
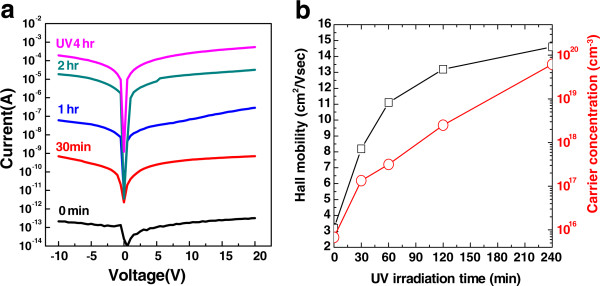
**Electrical properties such as conductivity and Hall mobility and carrier concentration of a-IGZO thin films.** Electrical properties such as **(a)** conductivity and **(b)** Hall mobility and carrier concentration of a-IGZO thin films as a function of UV irradiation time (0, 30, 60, 120, and 240 min). As UV irradiation time increases, electrical properties increase linearly owing to permanent optical doping.

Figure 2 shows the *I*-*V* curves, Hall mobility, and carrier concentration for a-IGZO thin films, depicting the dependence of their electrical properties on UV irradiation time in an air atmosphere; the films were deposited on a glass substrate at an Ar:O_2_ partial pressure ratio of 90:10 at room temperature. By comparing the electrical properties of a-IGZO thin films at various UV irradiation times, we confirmed that the electrical conductivity increased up to nine orders of magnitude proportionally to UV irradiation time, although the initial electrical properties of the thin films were identical. According to Hall measurement result at the room temperature on UV-irradiated a-IGZO film, it was confirmed that electrical properties such as Hall mobility and carrier concentration of the a-IGZO film also increase as UV irradiation time increases. In particular, the Hall measurement on 4-h UV-irradiated a-IGZO film revealed its metallic electrical properties such as the carrier concentration at 1.6 × 10^13^ cm^-2^, resistivity at 4.6 × 10^-3^ Ω∙cm, and Hall mobility at 14.6 cm^2^/Vs at room temperature. Since the Hall measurement was impossible for the as-deposited a-IGZO due to its highly insulating property, this is equivalent to the insulator-to-metal conversion of amorphous oxide by UV irradiation. This UV irradiation effect is permanently maintained since the time-dependant Hall measurement revealed no significant change in electrical properties over a 4-week air-aging test [see Additional file 1: Figure S1 for air-aging time-dependant Hall measurement data], and this implies that UV irradiation on a-IGZO more possibly leads to an irreversible change in chemistry acting as chemical doping to alter the electrical property of a-IGZO significantly. Therefore, the in-depth investigation on surface chemistry upon UV irradiation was performed by XPS analysis in the next section.

**Figure 3:**
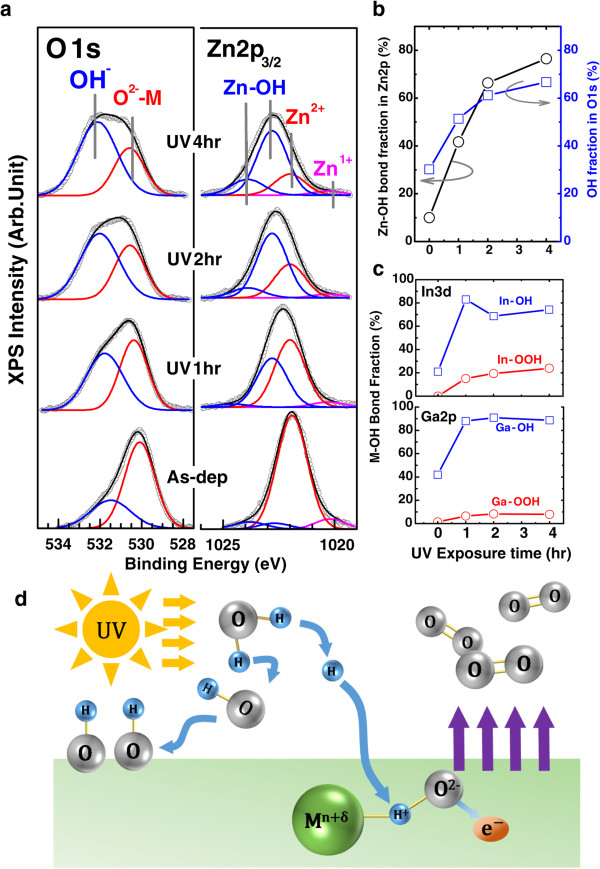
**Chemical binding states, bond fraction evolutions of XPS spectra, and UV/air photochemical H-doping scheme. (a)** O1s and Zn2p_3/2_ XPS spectra of the as-deposited a-IGZO with different UV/air exposure time and their Gaussian peak deconvolution. In the O1s XPS spectra, two chemical binding states were resolved: (1) O^2-^-M fully oxidized states (530.5 eV) and (2) OH (532 eV). For Zn ions, three types of binding states were extracted in each metal ion: (1) O-deficient Zn^1+^ equivalent to O-vacancy, (2) Zn-O in the fully oxidized state (Zn^2+^), and (3) Zn bonded to OH group. The bond fraction evolutions of **(b)** Zn-OH in Zn2p and OH in O1s and **(c)** In-OH [1]/[2] ([1] is for low and [2] is for high binding energy) and Ga-OH [1]/[2] as a function of UV exposure time. **(d)** Scheme of UV/air photochemical H-doping in a-IGZO.

XPS analyses were performed to investigate the origin of HECL in terms of the change in UV-assisted surface chemistry. Figure 3 shows narrow XPS scan spectra of O1s and Zn2p_3/2_ chemical binding states in the as-deposited and UV-irradiated a-IGZO. In the O1s XPS spectrum, two chemical binding states were resolved: (i) O-metal (530.5 eV) and (ii) OH-M (532 eV) binding states (where M represents In, Ga, and Zn); these binding states agree with those reported in many previous reports [16,17]. For Zn ions, three types of binding states were observed: (i) O-deficient Zn^1+^ (1,020 eV), (ii) Zn^2+^ in its full oxidation state (1,021.7 eV), and (iii) Zn-OH-related binding states (Zn-OH at 1,022.8 eV and Zn-OOH at 1,023.9 eV). In and Ga ions were also found to display the identical O- and OH-related binding states [see Additional file 1: Figure S2 for In3d and Ga2p spectra]. Each Zn2p binding state was consistent in the O1s and metal XPS spectra, except O-deficient Zn^1+^ that originates from the O-vacancy defect in the local metal-oxygen-bonding environment. The unsaturated O binding state in O-vacancy was not well resolved in the O1s spectra, while Zn^1+^ binding states were clearly resolved in the Zn2p spectra.

UV-irradiated a-IGZO surfaces showed significant changes in the metal-OH states. The metal-OH bond fractions normalized to the total areal intensity in each metal ion increase with increase in UV irradiation; this trend corresponds to an increase in the OH bond fraction in the O1s XPS spectra corresponding to UV irradiation. The chemical reason for higher binding energies of Zn-OH-related bonds is due to the higher group electronegativity of OH compared to O. Although the group electronegativity of OH varies at 2.3 to 3.9 due to the lone pair *π* electrons with the remainder of the molecule, Zn-OH configuration is subject to the higher group electronegativity of OH than that of O (3.44) at least since the binding energy of Zn-OH is higher than Zn-O in Zn2p spectra.

The origin of significantly increased OH bond of a-IGZO upon UV irradiation is related to the UV/air photochemistry. As described in the reaction scheme (Figure 3d), due to UV photon energies used in this study, abundant excited OH and H radicals are produced by photochemical water dissociation and react with the a-IGZO surface. Among those radical species, H is most effective for surface reaction due to the fast diffusion near the a-IGZO surface. On the other hand, UV light itself is well known to break O-metal bonds to create O-vacancy sites in metal-oxygen bonds like Zn-O by the photocarrier-induced O desorption [18]. This provides the basis for substitutional H incorporation in addition to interstitial incorporation. Therefore, the proposed a-IGZO surface reaction scheme under UV/air irradiation can be regarded as H incorporation to (1) O-vacancy substitutional sites and (2) interstitial sites (due to a small size of H) and finally appears as OH bonds. It was suggested that H incorporation into a-IGZO always results in surface OH bond formation where conversion of H atoms to H^+^ ions (i.e., donor ions) increases the carrier concentration greatly as expressed in the following equation: H(ads) + Zn^2+^ + O^2-^ (in a-IGZO) → H^+^(in lattice) + Zn^2+^ + O^2-^ + e^-^ → OH^-^ (in a-IGZO) + Zn^2+^ + e^-9^. Here, similar reactions for In and Ga in addition to Zn can be considered. In order words, H atom donates excess electrons while being the positively ionized donor ion in a-IGZO and is finally converted to - OH or - O^2-^H^+^. This effect is also confirmed in H-doped ZnO in the view of electronic bond state, and interestingly, it was explained that an excess electron initially occupies the high-lying Zn (4s)-H (1s) antibonding molecular orbital (MO) state above conduction band (CB) edge of ZnO and deexcites to CB minimum states to be free carriers [19]. Similar chemical doping mechanism and coupling of excess electrons with In/Ga (5s)-H (1s) is highly expected. Since electrons are confined in s-orbital character MO state positioned at the edge of CB minimum, the carrier mobility should be higher than electrons trapped at the d-band dominant deep donor level of O-vacancy. The OH formation of a-IGZO upon H-induced n-type doping has been experimentally demonstrated in previous studies using thermal anneal and ion implantation [20]. However, the overall doping effect with such doping methods is not so significant, and this was believed due to the doping effect compensation by the complicated interaction between loosely bound O and H subject to the thermal diffusion throughout the bulk. Nevertheless, UV-induced H-doping in this study reveals the remarkable effect of the insulator-to-metal transition. We regard that the key mechanism for this distinguishable from other H-doping technique is related to double simultaneous photochemical reactions; O-vacancy creation and H incorporation, synergistically forming highly OH-rich a-IGZO surface not throughout the bulk. The surface localized H incorporation is inherent property of UV photochemistry done at room temperature free of significant thermal diffusion or high energy ion penetration. Also, we found no change in the bulk optical bandgap by spectroscopic ellipsometry [see Additional file 1: Figure S3 for optical absorption data]. These unchanged optical properties even after UV irradiation are due to weak light absorption in the surface OH region and should have been altered if this is the bulk doping effect since bulk zinc hydroxide (ZnOH_2_) has at least 1 eV higher bandgap than ZnO [21].

**Figure 4:**
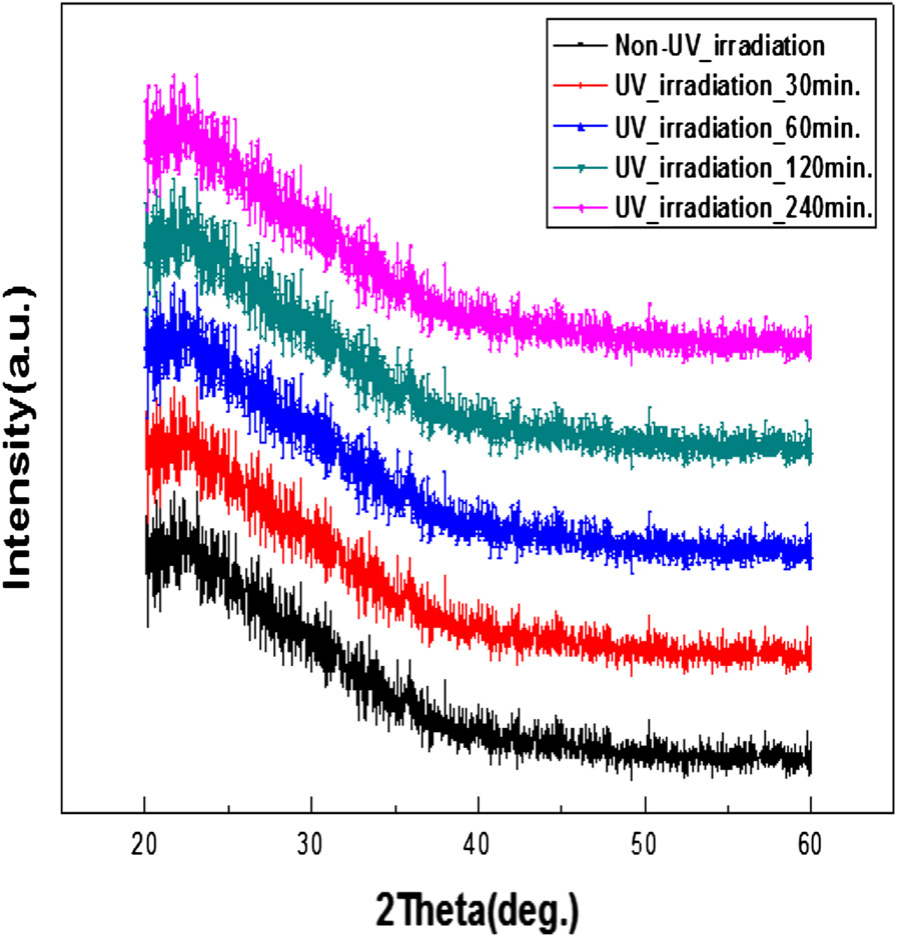
**XRD pattern analysis for a-IGZO thin film phases subjected to UV irradiation for different times.** Regardless of UV irradiation time, all a-IGZO thin films exhibited an amorphous phase. This observation confirmed that electrical conductivity changes after UV irradiation cannot be attributed to the formation of a crystalline structure.

**Figure 5:**
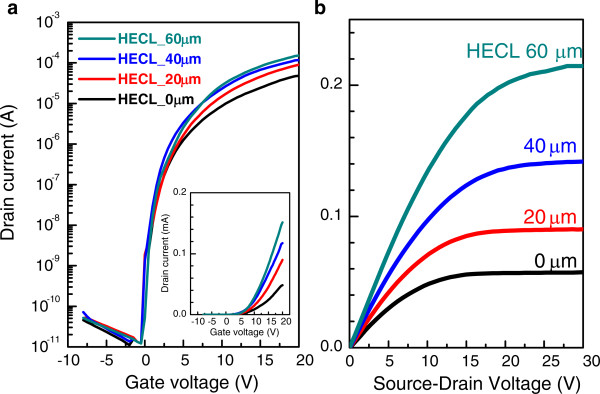
**Transfer characteristics and *I***_**ds**_**-*V***_**ds **_**curves of DAL TFTs with varying HECL length. (a)** Transfer characteristics and **(b)***I*_ds_-*V*_ds_ curves of DAL TFTs with varying HECL length. On-current increased monotonically as the HECL length increased within the same channel. The inset figure is the linear scale curve. This observation confirmed that superior electrical properties are obtained with an increase in the on-current and no change in the off-current, as a semiconducting gap with a width of 10 μm exists between source/drain and HECL.

To investigate whether such an electrical conductivity change due to HECL formation was free of UV-induced bulk phase change in the a-IGZO thin films, X-ray diffraction measurements were performed to analyze the crystallinity of thin films after UV irradiation for various times. Figure 4 shows the XRD patterns of a-IGZO thin films deposited at room temperature using a sputtering method and UV irradiation for various times. The XRD patterns do not indicate a crystalline peak regardless of the UV irradiation time; thus, it can be stated as XRD amorphous. Nevertheless, XRD is unable to detect a nanocrystallinity (nc) at the length of ordering scale less than approximately 4 nm [22]. To investigate the effects of HECL formation for TFT performance, we compared the transfer characteristics of TFTs according to changes in HECL length in the active layer (Figure 5). Each transfer curve shows the results measured under the same conditions of *V*_ds_ =18 V and TFT drain current in the saturation region (*V*_ds_ ≥ *V*_gs_ - *V*_th_) as represented by Equation 1

(1) $$I_{\text{ds}}=\frac{W\mu_{\text{sat}}C_{i}}{2L}{\left(V_{\text{gs}}-V_{\text{th}}\right)}^{2}$$

where *μ*_sat_ is the saturation mobility, *V*_th_ is the threshold voltage, *L* is the channel length, *W* is the channel width, and *C*_*i*_ is the electrostatic capacity of a dielectric layer per unit area [23]. The systematic increase in the drain current level is observed with UV irradiation time, and this trend is also confirmed in *I*_ds_-*V*_ds_ curve (Figure 5b).

**Figure 6:**
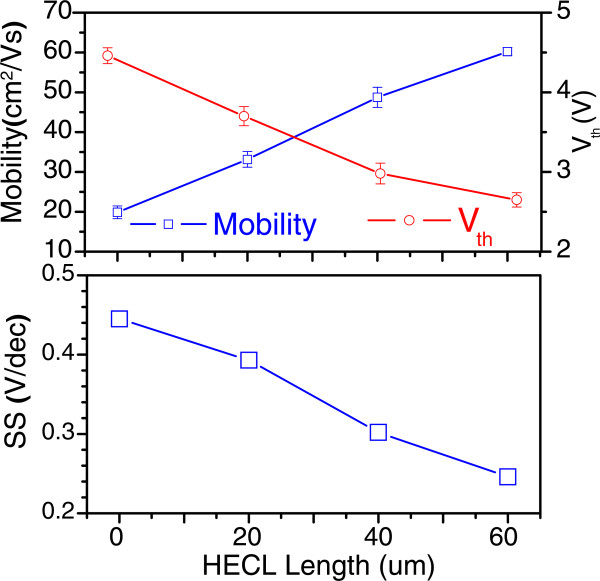
**Changes in saturation mobility and threshold voltage of a-IGZO according to HECL length.** As the HECL length increases, carrier concentration increases within the channel path, which results in a linear increase in carrier concentration. In contrast, the TFT is turned on at a lower gate voltage as the HECL length increases.

Figure 6 shows changes in the threshold voltage, saturation mobility, and subthreshold swing (SS) with the HECL length, which are derived by taking the square root of Equation 1 $\sqrt{I_{\text{ds}}=}{\left(W\mu_{\text{sat}}C_{i}/2L\right)}^{1/2}.\left(V_{\text{gs}}-V_{\text{th}}\right)$. The TFT without an HECL embedded in a channel path exhibited a saturation mobility of 19.9 cm^2^/Vs, threshold voltage of 4.5 V, and SS of 0.45 V/decade. In contrast, the TFT with a 60-μm-length HECL embedded in the channel path exhibited superior switching characteristics such as a saturation mobility of 60.2 cm^2^/Vs, threshold voltage of 2.7 V, and SS of 0.25 V/decade. Therefore, the saturation mobility increased up to a factor of 3 by inserting a 60-μm-length HECL compared to that in the normal TFT. In a previous study on mobility enhancement [24], a carrier concentration increase in the active layer led to an increase in both on- and off-currents, which eventually caused an increase in the TFT leakage current. However, in the case of a DAL TFT with an HECL embedded in the active layer in this study, only the on-current was increased without any change in the off-current, as shown in Figure 5. It is believed that the partial embedding of HECL within the 80-μm-long active layer induced the formation of a semiconducting a-IGZO gap with a width of at least 10 μm between source/drain and HECL. Therefore, the mobility could be enhanced without any increase in the leakage currents. In a previous study on the carrier mobility in a-IGZO, it was reported that there is a direct relationship between the carrier concentration and mobility [25,26]. In general crystalline Si semiconductors, the mobility decreases owing to increased scattering as the carrier concentration increases; in contrast, in a-IGZO, the mobility increases monotonically with carrier concentration owing to the disorder in the amorphous structure. Our experiment confirmed that as the HECL length increases, the carrier concentration also increases, leading to a linear increase in the carrier mobility as well. In the circuit point of view, the proposed TFT structure is indeed equivalent to two serially connected TFTs (i.e., 10-μm-long active layer for each TFT) via the middle HECL layer (i.e., 60-μm-long conducting channel layer). Therefore, the true mobility in undoped a-IGZO channel remains the same. Nevertheless, based on Hall measurement, it is clear that the H-doped a-IGZO HECL layer has a much higher bulk carrier mobility (at 14.6 cm^2^/Vs) than that in as-deposited a-IGZO (below detection limit at approximately 0.01 cm^2^/Vs). This contributes to the ‘mean-field mobility’ improvement as a whole. Typically, upon the formation of imbedded metal conducting layer in a-IGZO TFT, a heterogeneous interface of metal/a-IGZO is highly possible to invoke additional problems such as new contact resistance, interfacial chemical mixing, charge trapping, etc. Our approach using homogeneous a-IGZO conducting channel using H-doping is free of those problems.

In addition to the mobility enhancement, it was confirmed that TFTs can be turned on at a lower gate voltage when the carrier concentration increases, and the threshold voltage is gradually lowered owing to the increased length of embedded HECL within a channel path. The relationship between the carrier concentration and threshold voltage has been reported in a previous study [4]. The concurrent changes in crucial TFT parameters such as saturation mobility and *V*_th_ are closely related to chemical changes of a-IGZO by UV-induced H-doping. As shown in Figure 3d, the high OH fraction is equivalent to a surface H-doping effect or hydroxide formation on a-IGZO. Thus, the surface potential of an HECL led by UV photochemistry leads to a relatively negative *V*_th_ shift in TFT devices.

Additionally, it can be found that intensity of the O-deficient Zn^1+^ (1,020 eV) peak is decrease with in UV irradiation, as shown in Figure 3. It means that O-vacancy defects acting trap site in the local metal-oxygen-bonding environment decreased. Therefore, increase of HECL length leads to the decrease in the density of trap site at the whole active channel layer. In general, SS value is closely related to the density of trap site in active layer of TFTs [27]. Hence, as the HECL length increases, the SS value of the device also improves.

## Conclusions

In this study, we fabricated DAL TFTs via a new approach called permanent photochemical doping to enhance the mobility of existing bottom-gate-structure TFTs. Different from existing DAL TFT mobility enhancement methods, the structure resulting from this approach employs the same phase and material; thus, degradation in electrical properties due to physical interface formation or band offset does not occur. The chemical origin of permanent optical doping in HECL was found as UV photochemistry induced intensive and uniform surface H-doping of a-IGZO proved by XPS. Moreover, the effects of changes in the HECL length on the channel path were investigated. The obtained results showed that the mobility increased monotonically as the length of the embedded HECL increased. This result was attributed to the carrier concentration increase in the active layer channel area triggered by changes in the HECL length. Finally, the highest mobility of 60.2 cm^2^/Vs was obtained when a 60-μm-long HECL was embedded in an 80-μm-long channel path. This observation confirmed that an HECL length 75% of the total active layer length results in high-performance characteristics for materials with 200% improvement in mobility: a threshold voltage of 2.7 V, SS of 0.25 V/decade, and an on/off ratio of 10^7^. Moreover, the leakage current level was maintained at 10^-11^ A. All these significant improvements in a-IGZO TFTs are explained by the effective n-doping in HECL by UV-induced H-incorporation on the surface, confirmed by OH-rich surface of a-IGZO. Surface H-doping results in a high electron donation in the HECL even for amorphous a-IGZO without any heat treatment to improve crystallinity and dopant activation. Therefore, owing to its great simplicity, the comprehensive performance improvement achievable, and stability, the doping technique proposed herein can be effectively utilized for large-scale a-IGZO TFT arrays employed in flat-panel displays. The applicability of the H-doping technique proposed in this study can be extended if it becomes possible to carry out selective UV irradiation along with *in situ* a-IGZO deposition through improvements in process equipment in the future.

## Competing interests

The authors declare that they have no competing interests.

## Authors’ contributions

SJ, HS, and DC designed the experiments. SJ, MK, and SL carried out the experiments and tested the devices. SJ, HS, and DC analyzed the data and wrote the manuscript. All authors read and approved the final manuscript.

## Supplementary Material

Additional file 1 **Dual active layer a-IGZO TFT via homogeneous conductive layer formation by photochemical H-doping. Figure S1.** Hall measurement results (carrier concentration and Hall mobility) as a function of air aging-time. **Figure S2.** In3d and Ga2p XPS data of the as-deposited and UV-exposed a-IGZO with Gaussian peak deconvolution. **Figure S3.** Absorption coefficient spectra for the as-deposited and UV-irradiated a-IGZO taken by spectroscopic ellipsometry analysis. Click here for file
