# Supplementary material for: Dual active layer a-IGZO TFT via homogeneous conductive layer formation by photochemical H-doping
Source: Nanoscale Res Lett. 2014 Nov 18;9(1):619. doi: 10.1186/1556-276X-9-619 (PMC4244097; doi:10.1186/1556-276X-9-619)
Supplement: Additional file 1 — Dual active layer a-IGZO TFT via homogeneous conductive layer formation by photochemical H-doping. Figure S1. Hall measurement results (carrier concentration and Hall mobility) as a function of air aging-time. Figure S2. In3d and Ga2p XPS data of the as-deposited and UV-exposed a-IGZO with Gaussian peak deconvolution. Figure S3. Absorption coefficient spectra for the as-deposited and UV-irradiated a-IGZO taken by spectroscopic ellipsometry analysis. [file 1556-276X-9-619-S1.docx]

Supplemental Information

Dual-Active-Layers a-IGZO TFT via Homogeneous Conductive Layer Formation by Photochemical H-doping

*Seung-Ki Jeong, Myeong-Ho Kim, Sang-Yeon Lee, Hyungtak Seo and Duck-Kyun Choi*

1. **Time dependent doping stability test**

**Fig. S1. Hall measurement results (carrier concentration and hall mobility) as a function of air aging-time.** The a-IGZO/glass samples was maintained in the lab air since fabricated. No significant changes in electrical parameters such as carrier concentration and hall mobility was found even after 4 week air aging. This confirms that H doping by UV irradiation is permanent and not due to the transient photocurrent, which is typically subject to several hr recovery due to photocarrier detrapping from defect states.

1. **XPS data of In3d and Ga2p**

**Fig. S2. In3d and Ga2p XPS data of the as-deposited and UV-exposed a-IGZO with Gaussian peak deconvolution.** Similar to the Zn ion, three types of binding states were observed in each metal ion: (1) O-deficient In^2+^ and Ga^2+^ equivalent to the O-vacancy; (2) In- and Ga-O in the full oxidation state (In^3+^ and Ga^3+^); and (3) OH-related In/Ga bond (denoted as In/Ga-OH [1] and In/Ga-OH [2] or possibly, In/Ga-O-OH).

1. **Absorption data of a-IGZO before and after UV-irradiation**

**Fig. S3. Absorption coefficient spectra for the as-deposited and UV-irradiated a-IGZO taken by spectroscopic ellipsometry analysis.** The inset figure shows the magnified spectra at 3~4 eV of photo energy. All samples reveals the absorption onset at ~ 3.4 eV and no significant change in typical absorption edge. This data suggests UV-irradiation does not incur the bulk optical properties in consistent with XPS results showing the strong H-doping effect only at the surface region.
